# Structural and functional insights into the interaction and targeting hub TMD0 of the polypeptide transporter TAPL

**DOI:** 10.1038/s41598-018-33841-w

**Published:** 2018-10-23

**Authors:** Christoph Bock, Frank Löhr, Franz Tumulka, Katrin Reichel, Julia Würz, Gerhard Hummer, Lars Schäfer, Robert Tampé, Benesh Joseph, Frank Bernhard, Volker Dötsch, Rupert Abele

**Affiliations:** 1 Institute of Biochemistry, Biocenter, Goethe University Frankfurt, Max-von-Laue Str. 9, 60438 Frankfurt am Main, Germany; 2 Institute of Biophysical Chemistry & Center for Biomolecular Magnetic Resonance, Goethe University Frankfurt, Max-von-Laue Str. 9, 60438 Frankfurt am Main, Germany; 3 Institute of Physical and Theoretical Chemistry, Goethe University Frankfurt, Max-von-Laue Str. 9, 60438 Frankfurt am Main, Germany; 4 Department of Theoretical Biophysics, Max Planck Institute of Biophysics, Max-von-Laue Str. 3, 60438 Frankfurt am Main, Germany; 5 Lehrstuhl für Theoretische Chemie, Ruhr-University Bochum, 4780 Bochum, Germany

## Abstract

The ATP-binding cassette transporter TAPL translocates polypeptides from the cytosol into the lysosomal lumen. TAPL can be divided into two functional units: coreTAPL, active in ATP-dependent peptide translocation, and the N-terminal membrane spanning domain, TMD0, responsible for cellular localization and interaction with the lysosomal associated membrane proteins LAMP-1 and LAMP-2. Although the structure and function of ABC transporters were intensively analyzed in the past, the knowledge about accessory membrane embedded domains is limited. Therefore, we expressed the TMD0 of TAPL via a cell-free expression system and confirmed its correct folding by NMR and interaction studies. In cell as well as cell-free expressed TMD0 forms oligomers, which were assigned as dimers by PELDOR spectroscopy and static light scattering. By NMR spectroscopy of uniformly and selectively isotope labeled TMD0 we performed a complete backbone and partial side chain assignment. Accordingly, TMD0 has a four transmembrane helix topology with a short helical segment in a lysosomal loop. The topology of TMD0 was confirmed by paramagnetic relaxation enhancement with paramagnetic stearic acid as well as by nuclear Overhauser effects with c6-DHPC and cross-peaks with water.

## Introduction

ATP-binding cassette (ABC) proteins represent the largest family of primary active membrane transport proteins^1^. Although some members of the superfamily represent soluble proteins involved in DNA repair as well as mRNA translation, most members constitute transporters translocating a large spectrum of different solutes across cellular membranes driven by the energy of ATP hydrolysis^2^. ABC transporters are found in all kingdoms of life operating as importer, exporter, channel, or regulator. Their dysfunctions have severe clinical impacts including cystic fibrosis, diabetes, multidrug and antibiotic resistance, as well as Alzheimer’s disease^3^. ABC transporters have a modular architecture composed of two highly conserved nucleotide binding-domains (NBD) and two transmembrane domains (TMD), which are more diverse than the NBDs. Eukaryotic ABC transporters form full-size transporters, with all four domains located on one polypeptide chain, or half-size transporters, composed of a TMD and an NBD. The TMDs of eukaryotic ABC transporters are composed of 2 × 6 transmembrane helices (TMHs). However, some members of the ABCB and ABCC subfamily contain an additional membrane embedded domain named TMD0. Members of the ABCC family represent full-size transporters, some of which harbor an extra five TMHs containing N-terminal TMD0^4^. Dependent on the transporter, the TMD0 is essential for subcellular trafficking, protein processing, and interaction with other proteins as in the case of SUR1, which binds to the K^+^-channel Kir6.2 via its TMD0. In the ABCB subfamily, TMD0s are only found in the half-size transporters associated with antigen processing 1 (TAP1) and 2 (TAP2) (ABCB2/3), ABCB6, and TAP-like (TAPL, ABCB9), and are therefore resulting in two TMD0s per functional transport complex. For all ABCB half-size transporters, the TMD0 is not essential for dimerization and transport activity. The five TMHs comprising TMD0 of ABCB6 are required for subcellular targeting. As the full transporter, TMD0 alone is correctly localized in lysosomes whereas coreABCB6 shows mislocalization to the plasma membrane^5^. In the heterodimeric complex TAP, composed of TAP1 and TAP2, the TMD0s interact with tapasin via a conserved salt bridge in the center of the endoplasmic reticulum membrane^6^. This interaction is crucial for the peptide loading complex (PLC) assembly and an efficient major histocompatibility complex class I antigen presentation^7^.

**Figure 1:**
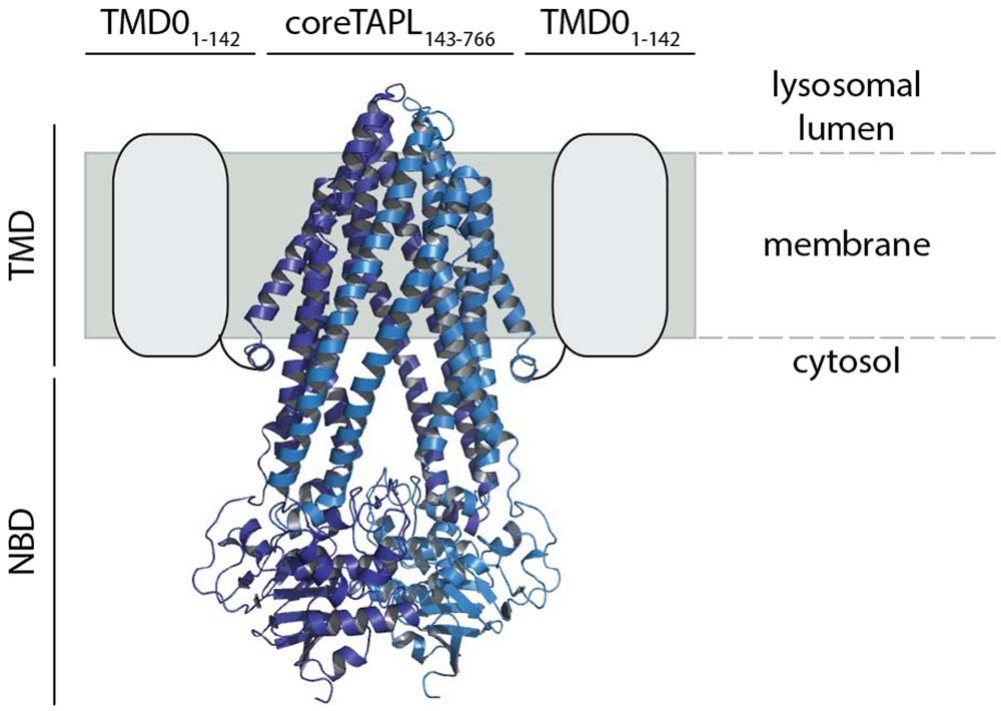
Architecture of homodimeric TAPL complex. Functionally, the half-size transporter TAPL can be divided in an N-terminal TMD0 (grey box), which is essential for lysosomal targeting and the interaction with LAMP-1/2, and coreTAPL (light and dark blue) composed of six TMHs and a C-terminal, cytosolic NBD. CoreTAPL forms dimers and is fully active in peptide transport but mislocalized to the plasma membrane. CoreTAPL was modeled with SWISS-MODEL on the X-ray structure of ABCB10 (PDB ID: 4YAT)^40,41^.

In our studies we focused on the TMD0 of TAPL, which translocates cytosolic polypeptides (6-mer up to 59-mer) into the lumen of lysosomes^8^. TAPL seems to be an evolutionary ancient transporter since orthologues are found in vacuoles and lysosome like compartments in plants and invertebrates^9,10^. In mammals, TAPL shows a broad tissue distribution with strong expression in testis and brain^11^. Moreover, high amounts of TAPL are found in dendritic cells and macrophages^12^. The homodimeric half-transporter TAPL comprises two functional units (Fig. 1): (1) coreTAPL (residue 143 to 766), consisting of two conserved NBDs and 2 × 6 TMHs, is fully functional in peptide transport; (2) the N-terminal transmembrane domain TMD0 (residue 1 to 142). TMD0 harbors the lysosomal targeting signal since coreTAPL is mistargeted to the plasma membrane whereas cellular trafficking to lysosomes is rescued by co-expression with TMD0^13^. Beyond trafficking, TMD0 displays an interaction platform with the lysosomal associated membrane proteins LAMP-1 and LAMP-2B. This interaction extends the half-life of TAPL by a factor of five^14^. While cryo electron microscopy (EM) structures from TMD0s of the ABCC family exist^15,16^, none of the structures of the ABCB family’s extra transmembrane domain has been revealed. To structurally characterize TMD0 by solution nuclear magnetic resonance (NMR) spectroscopy, an *Escherichia coli* based cell-free (cf) expression protocol was established, in which an optimized TMD0 variant (Fig. S1) was synthesized in the absence of detergent and refolded during purification to yield a stable α-helical protein^17^. After adjustment of buffer, detergent, ionic strength and temperature transverse relaxation optimized spectroscopy (TROSY) NMR spectra with sufficient resolution and homogeneity were obtained^17^.

Here, we analyzed the cf variant of TMD0 in respect of correct folding and oligomerization and compared it with wt-TMD0. We demonstrated a similar behavior of cf- and wt-TMD0 with regard to their interaction with coreTAPL and oligomerization when expressed in human cells. Cf expressed TMD0 forms dynamic dimers and specifically interacts with coreTAPL. A series of HN-detected 3D triple resonance NMR spectra of selectively isotope labeled TMD0 allowing full backbone and partial side chain assignment revealed a four TMH topology with a short helical segment in a lysosomal loop. The topology of TMD0 was confirmed by paramagnetic relaxation enhancement (PRE) and by nuclear Overhauser effects (NOEs).

## Results

### Functionality of cell-free expressed TMD0

**Figure 2:**
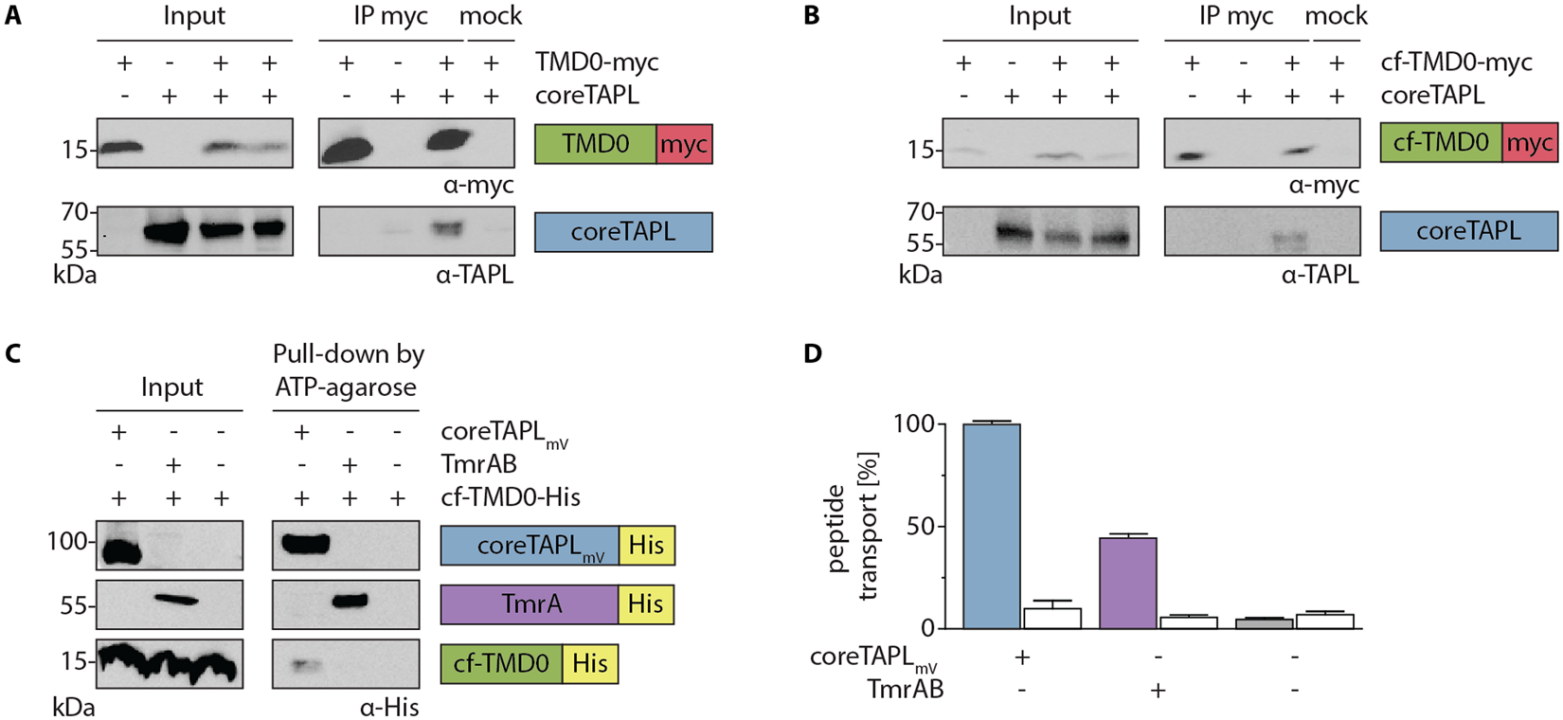
Cf expressed TMD0 is correctly folded. (**A**,**B**) Interaction of coreTAPL with TMD0. HEK 293 T cells were transiently transfected with coreTAPL and TMD0-myc (**A**) or cf-TMD0-myc (**B**). After solubilization with 1% digitonin (input), co-immunoprecipitation with myc-tag specific antibody (IP myc) or isotope control (mock) was performed and analyzed by immunoblotting. Input represents 1/25 aliquot of the volume used for immunoprecipitation. Original, uncropped immunoblots are shown in supplementary information. (**C**) To probe correct folding of cf expressed TMD0, cf-TMD0-His was reconstituted into proteoliposomes alone or together with coreTAPL_mV_ or TmrAB (input). After solubilization with 2% digitonin, coreTAPL_mV_ or TmrAB were pulled-down with ATP-agarose and analyzed by immunoblotting using a His-tag antibody. Input represents 1/20 aliquot of the volume used for pull-down. Original, uncropped immunoblots are shown in supplementary information. (**D**) To confirm functional reconstitution, proteoliposomes containing only cf-TMD0-His or cf-TMD0-His and coreTAPL_mV_ or TmrAB were incubated in the presence (filled bars) or absence (open bar) of ATP (3 mM) with peptide (3 µM) for 15 min at 37 °C. Transport assay was performed in triplicates and normalized, error bars indicate SD.

Before doing a biochemical and structural analysis of the TMD0 variants, we tested them for structural integrity since the used constructs contain N-terminal mutations R_2_K and W_4_Y for optimized translation initiation in cf expression, substitutions of all four cysteines to alanine, and a C-terminal tag for purification. All constructs used in this study are summarized in Fig. S1. TMD0 shows no enzymatic activity, therefore, we addressed correct folding by the interaction of TMD0 with coreTAPL. Human embryonic kidney (HEK) 293 T cells were transiently transfected with expression constructs of TMD0-myc or cf-TMD0-myc alone or together with coreTAPL and lysed after 48 h with the mild detergent digitonin. CoreTAPL is co-immunoprecipitated with wt- as well as cf-TMD0. Therefore, the N-terminal mutations and cysteine substitutions in cf-TMD0 do not interfere with the coreTAPL interaction, indicating correct folding (Fig. 2A,B).

Next, we analyzed correct folding of cf expressed TMD0. Cf-TMD0-His was expressed in an *E. coli* based cf expression system in the precipitation mode, in which membrane proteins are synthesized in the absence of any detergents or lipids and therefore tend to precipitate^17^. Proteins were solubilized by 1% 1-myristoyl-2-hydroxy-*sn*-glycerol-3-[phospho-*rac*-(1-glycerol)] (LMPG) and bound to Ni^2+^-nitrilotriacetic acid-agarose. Protein was refolded by exchanging stepwise LMPG to the mild detergent 1,2-dihexanoyl-*sn*-glycero-3-phosphocholine (c6-DHPC) during washing (Fig. S2). To test the structural integrity of cf-TMD0-His, the interaction of cf-TMD0-His with coreTAPL_mV_ was investigated. CoreTAPL_mV_ was expressed in *Pichia pastoris*, solubilized, and purified in dodecyl-β-D-maltoside (DDM). Since complex formation in detergent mixture of DDM and c6-DHPC was not practicable, cf-TMD0-His was reconstituted in the presence and absence of coreTAPL_mV_ (molar ratio 1:1) into liposomes composed of *E. coli* polar lipids and 1,2-di-(9-octadecenoyl)-*sn*-glycero-3-phosphocholine (DOPC) (Fig. 2C). As negative control, cf-TMD0-His was reconstituted under identical conditions with the peptide ABC transporter TmrAB from *Thermus thermophilus*. Subsequently, proteoliposomes were solubilized by digitonin followed by a pull-down with ATP-agarose, which binds to the NBDs of the ABC transporters. Cf-TMD0-His was precipitated only in the presence of coreTAPL_mV_ but not in its absence or presence of TmrAB. Functional reconstitution of coreTAPL_mV_ as well as TmrAB into liposomes was demonstrated by ATP-dependent peptide transport (Fig. 2D). The interaction of cf-TMD0-His and coreTAPL_mV_ got lost when DDM instead of digitonin was used to solubilize proteoliposomes (Fig. S3). In conclusion, cf expressed TMD0 specifically binds to coreTAPL and, consequently, is correctly folded.

### Oligomeric state of TMD0

**Figure 3:**
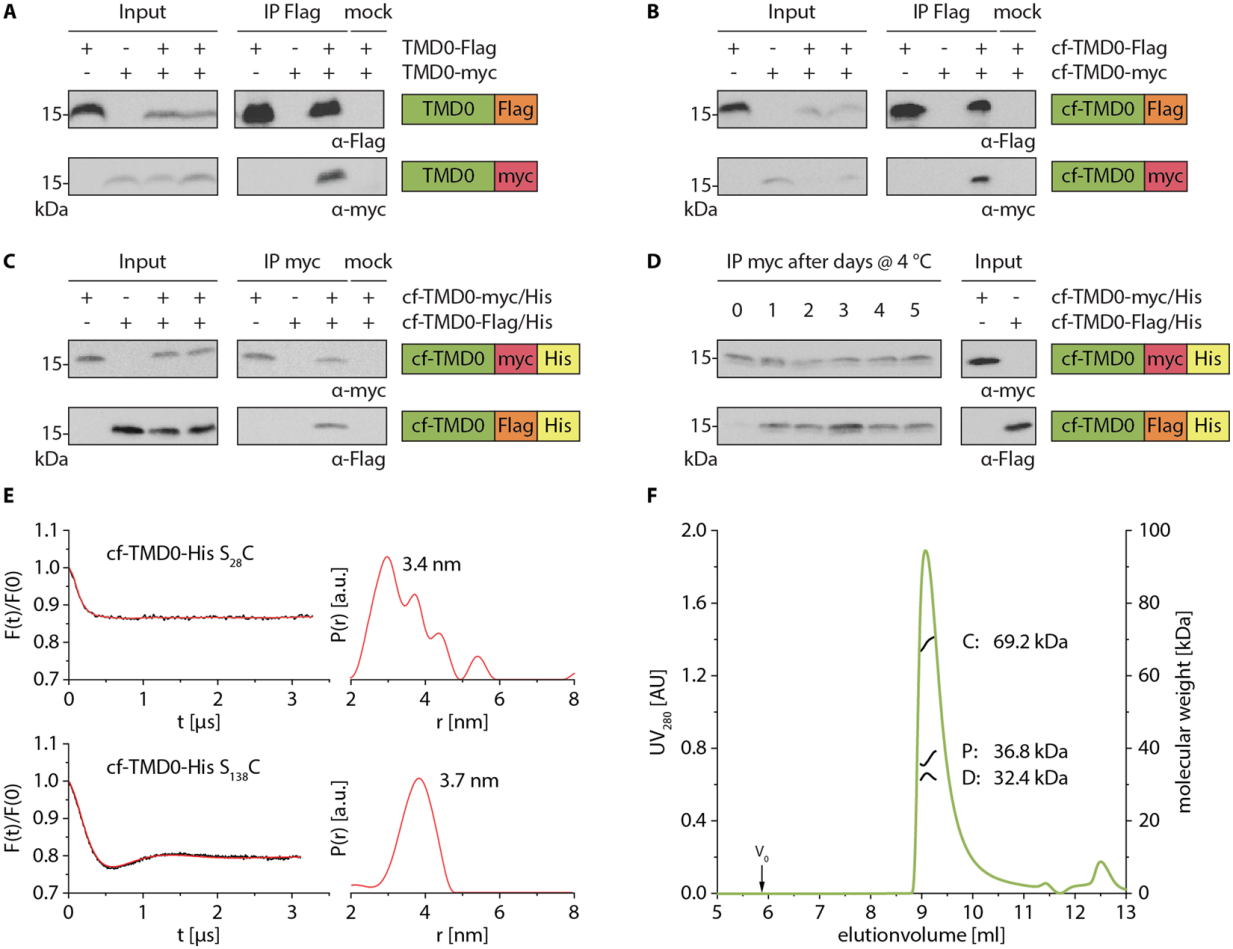
TMD0 oligomerization. (**A**,**B**) To test oligomerization of TMD0 in cellular environment, HEK 293 T cells were transiently transfected with wt **(A)** and cf variants (**B**) of TMD0 carrying a C-terminal Flag- or myc-tag. After solubilization with 1% digitonin, co-immunoprecipitation with Flag-tag specific antibody or isotype control (mock) was performed and analyzed by immunoblotting. Input represents 1/25 aliquot of the volume used for immunoprecipitation. Original, uncropped immunoblots are shown in supplementary information. (**C**) To analyze oligomerization of cf expressed TMD0, precipitates of separately produced cf-TMD0-myc/His and cf-TMD0-Flag/His were mixed, solubilized in LMPG, purified and reconstituted in c6-DHPC. Single or co-purified TMD0 variants (input) were immunoprecipitated by myc-tag specific antibody or isotype control and analyzed by immunoblotting. Input represents 1/15 aliquot of the volume used for pull-down. Original, uncropped immunoblots are shown in supplementary information. (**D**) To probe the oligomerization dynamics, cf expressed TMD0 variants were separately purified and refolded (input). Cf-TMD0-myc/His and cf-TMD0-Flag/His were mixed in a 1:1 molar ratio and incubated at 4 °C up to five days. Exchange of subunits was analyzed by immunoprecipitation with myc-tag specific antibody. Input represents 1/15 aliquot of the volume used for pull-down. Original, uncropped immunoblots are shown in supplementary information. (**E**) Oligomerization of MTSL labeled cf-TMD0-His S_28_C and cf-TMD0-His S_138_C was investigated by PELDOR spectroscopy. PELDOR traces were background corrected (left), and corresponding distance distributions were calculated by Tikhonov regularization (right). (**F**) Dimer formation of cf-TMD0-His was determined by SEC-MALS. 400 µg of purified cf-TMD0-His were applied on a 15 ml TSK-Gel G3000 SWXL column (green line). Molecular weight distributions were calculated by three-detector method using Astra software and are depicted in black lines (C: protein-detergent conjugate, P: protein, D: detergent).

The rotational correlation time (τ_c_) of cf-TMD0-His in c6-DHPC was determined by a 2D version of the TROSY for rotational correlation time (TRACT) NMR experiment to 25 ns at 323 K indicating oligomer formation of cf-TMD0-His^17^. To address oligomerization of TMD0, we expressed by transient transfection of HEK 293 T cells wt- and cf-TMD0 containing a C-terminal Flag- or myc-tag. Cells were lysed by digitonin, and TMD0 variants were immunoprecipitated via Flag-tag antibody. TMD0-myc as well as cf-TMD0-myc was co-immunoprecipitated by an anti-Flag antibody with its Flag-tag containing counterparts. However, cf-TMD0-myc was not precipitated in the absence of Flag-tagged TMD0 nor by an unrelated antibody demonstrating a specific oligomerization of TMD0 in membranes (Fig. 3A,B). To explore oligomer formation of cf expressed TMD0, which is an essential knowledge for structural investigation by NMR, cf-TMD0-myc/His and cf-TMD0-Flag/His were separately cf expressed and subsequently mixed for solubilization by LMPG, purification, and refolding. Cf-TMD0-Flag/His was co-immunoprecipitated by an anti-myc antibody, with cf-TMD0-myc/His being present during purification and refolding. However, cf-TMD0-Flag/His was not precipitated in the absence of cf-TMD0-myc/His nor by an unrelated antibody substantiating oligomerization of cf expressed TMD0 (Fig. 3C). His-tag dependent oligomerization was excluded since cf-TMD0-myc co-expressed with cf-TMD0-His could be co-precipitated by Ni-NTA-agarose whereas cf-TMD0-myc in the absence of cf-TMD0-His was not precipitated (Fig. S4).

To analyze the dynamics of TMD0 oligomerization, an important parameter in structural analysis, we mixed separately purified and refolded cf-TMD0-myc/His and cf-TMD0-Flag/His. Subsequently protein mixtures were incubated at 4 °C or room temperature prior to immunoprecipitation with anti-myc antibody (Fig. 3D, Fig. S5). An exchange of subunits was already detectable after 24 h of incubation as cf-TMD0-Flag/His was co-immunoprecipitated with cf-TMD0-myc/His. Therefore, TMD0 assemblies are very dynamic.

We next examined the stoichiometry of TMD0 oligomers. For this purpose, single cysteines were introduced in cysteine-less cf-TMD0-His at position S_28_ and S_138_. The structural integrity of single cysteine mutants was confirmed by two-dimensional TROSY experiments (Fig. S6). Single cysteine mutants were labeled with MTSL, and labeling efficiencies of 95 to 100% were determined by continuous wave (cw) electron paramagnetic resonance (EPR) spectroscopy with free 1-oxyl-2,2,5,5-tetramethyl-Δ3-pyrroline-3-methyl (MTSL) as reference. Using pulsed electron-electron double resonance (PELDOR, also known as DEER) spectroscopy, mean interspin distances of 3.4 ± 0.8 nm and 3.7 ± 0.5 nm for cf-TMD0-His S_28_C and cf-TMD0-His S_138_C, respectively, were obtained. The modulation depth of the intermolecular contribution corrected PELDOR traces fit to a two spin system indicating a TMD0 dimer (Fig. 3E). To confirm the dimeric state of cf-TMD0-His, we applied static light scattering using a size exclusion chromatography coupled with a UV/Vis spectrometer, differential refractometer and multi-angle light scattering detector (Fig. 3F). This three-detector method allows the assignment of the molecular weight of membrane proteins surrounded by detergent molecules. Cf-TMD0-His eluted in a single peak. Protein detergent conjugate has an average molecular mass of 69.2 kDa, whereas the detergent micelle comprises 32.4 kDa and cf-TMD0-His 36.8 kDa, which is matching with the mass of the dimer of cf-TMD0-His of 35.1 kDa. To get insights into the dimerization interface of TMD0, we compared the binding of TMD0 to full length with the core complex of TAPL (Fig. S7). We transiently transfected HEK 293 T cells with TMD0-myc together with coreTAPL or full length TAPL and performed immunoprecipitations with an anti-myc antibody. Interestingly, we could co-immunoprecipitate coreTAPL but not the full length complex. This shows that TMD0 is forming dimers only in the absence of the core complex and that the dimerization site of TMD0 matches most likely with the site involved in coreTAPL binding.

### Backbone assignment and secondary structure prediction

**Figure 4:**
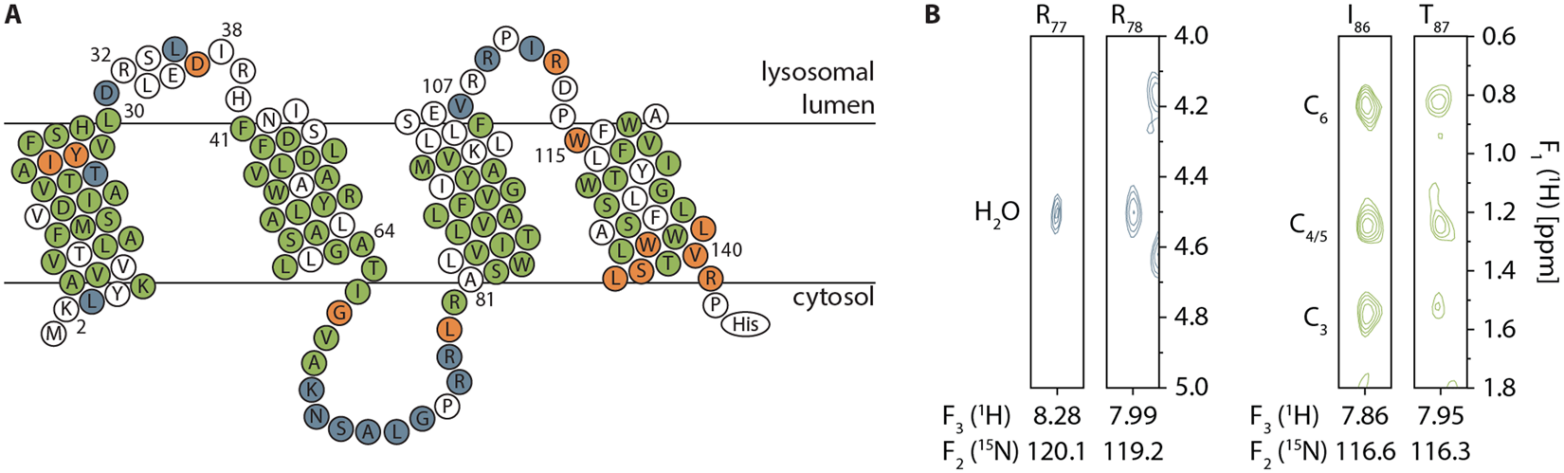
Secondary structure of TMD0. (**A**) Backbone chemical shifts derived from a series of HN-detected 2D triple resonance NMR spectra of cf-TMD0-His were used to generate a secondary structure model by TALOS-N. The numbers depict the first and last residue of each helix. Topology of cf-TMD0-His was derived from NOEs between ^2^H partially and ^15^N uniformly labeled cf-TMD0-His and protons of the hexanoyl side chains of c6-DHPC (green residues) and from cross-peaks with H_2_O (blue residues). Residues at the water micelle interface are involved in both restraints (orange residues). The N- and C-termini of cf-TMD0-His are oriented in the cytosol to fit within the overall topology with coreTAPL. (**B**) Exemplarily, exchange cross-peaks with H_2_O (left) and NOEs with c6-DHPC hexanoyl sidechains (right) in a 3D NOESY-BEST-[^15^N, ^1^H]-TROSY experiment (950 MHz, mixing time 300 ms) at 323 K of ^2^H partially and ^15^N uniformly labeled cf-TMD0-His (600 µM) are shown for an unstructured loop region (R_77_-R_78_) and an α-helical segment (I_86_-T_87_). Spectral sections are taken at F_2_ (^15^N) chemical shifts and centered (0.1 ppm) at the ^1^H chemical shifts (F_3_). ^1^H (F_1_) chemical shifts for H_2_O and C_3_, C_4/5_ and C_6_ of c6-DHPC hexanoyl side chain are indicated.

To get structural insights into TMD0 by solution NMR, cf-TMD0-His was expressed up to 1 mg per 1 ml cf reaction mix. Besides the high expression yield and velocity, the low isotopic scrambling is favorable. A recently invented extended labeling approach was applied, which allowed a nearly complete backbone assignment by recording a series of HN-detected 2D triple resonance NMR spectra^18^. HNCACB and 3D double-sensitivity enhanced CCH-TOCSY experiments enabled CB shift assignment and confirmed backbone assignment. An assignment of 97% for N, 99% for HN, 98% for CO, 98% for CA, 98% for HA and 66% for CB was achieved. The only problematic region was found between P_110_-P_114_, where the assignment was difficult due to the delimitating prolines lacking amide protons. All assigned chemical shifts were used to determine the secondary structure by TALOS-N (Fig. 4A). In TMD0, five α-helical segments but no β-strands or turns were identified. Four helices have a length between 24 and 29 residues with hydrophobic characteristics and are therefore assigned as TMH1 (K_2_ to L_30_), TMH2 (F_41_ to A_64_), TMH3 (A_81_ to V_107_) and TMH4 (W_115_ to V_140_). An additional loop helix (LH) (R_32_ to I_38_) is located between TMH1 and 2. All remaining residues form unstructured segments connecting the helices. The secondary structure assignment is supported by a network of sequential NOEs, which is restricted to structured regions (Fig. S8). Amide-amide NOEs up to two neighboring amino acids are most pronounced in TMHs and in the water-micelle interface. In loop regions, NOEs of only one neighboring amino acid are detected with weak intensities.

We explored the topology of TMD0 by PRE, in which the peak intensity is reduced due to the spatial proximity of the nucleus of interest to the paramagnetic center. Therefore, 1 mM of the fatty acid 16-DOXYL-stearic acid (16-DSA), containing a paramagnetic spin probe at C-16 of its aliphatic chain, was incorporated into detergent micelles. Peak intensities of residues within TMH1 to 4 are strongly reduced whereas residues in loop regions and LH are less effected, which is supporting the four TMH topology (Fig. S9).

The topology was also explored by NOEs between partially deuterated and uniformly ^15^N labeled cf-TMD0-His and the detergent c6-DHPC and cross-peaks with H_2_O in a 3D NOESY-BEST-[^15^N, ^1^H]-TROSY experiment. Exemplarily, cross-peaks with the water resonance for R_77_ and R_78_ present in loop 2 as well as NOEs of the hexanoyl side chains of c6-DHPC with I_86_ and T_87_ located in TMH3 are depicted (Fig. 4A,B). Overall, all TMHs showed strong interaction with the detergent c6-DHPC, whereas the unstructured segments mostly revealed accessibility to H_2_O supporting the model of four TMHs, which are surrounded by detergent. In the water-micelle interface of TMH1 and 4 contacts to c6-DHPC as well as H_2_O are observed. Interestingly, NOE signals between c6-DHPC and backbone amide protons were detected for a stretch of five residues (T_65_-A_69_) following the C-terminal end of TMH2. In summary, TMD0 is composed of five α-helical segments where the four longer helices are inserted in the membrane.

## Discussion

In this study, we biochemically and structurally characterized the targeting and interaction domain TMD0 of the lysosomal peptide transporter TAPL. The TMD0 construct designed for cf expression behaves as wt TMD0 in respect of expression yield in HEK 293 T cells, interaction with coreTAPL, and oligomerization. Moreover, cf-TMD0 variants produced by the precipitation based cf expression system can be functionally refolded as demonstrated by specific interaction with coreTAPL. In detergent solution, TMD0 forms a transient dimer. As derived from NMR studies, TMD0 contains four TMHs and one additional short helical segment oriented to the luminal site of lysosomes between TMH1 and TMH2.

Transient expression of wt- and cf-TMD0 in HEK 293 T cells resulted in similar amounts of protein. This is in strong contrast to the synthesis by the *E. coli* based cf expression, in which wt-TMD0 showed no expression, and only a substitution of the first six codons with an AU rich sequence resulted in significant amounts of protein^17^. It is reported that AU rich sequences in the region of translation start support strong translation by destabilizing inhibitory mRNA secondary structures^19^. However, this seems more significant for prokaryotic translation where ribosome binding and translation initiation are in closer proximity than in eukaryotes. Secondary structures in 5′-UTR of eukaryotes have more deleterious effects than in prokaryotes since the 43 S ribosomal initiation complex binds to the 5′cap and scans the mRNA until reaching an initiation codon^20^. Moreover, translation efficiencies of cf and in cell expression can differ drastically^21^.

Importantly, cf expressed TMD0 is functional and therefore correctly folded since it specifically interacts with coreTAPL as demonstrated by co-purification after reconstitution in proteoliposomes. Although such pull-downs are not quantitative, we assume that mostly all TMD0 molecules are correctly folded since only one stable conformation was observed by NMR spectroscopy. TMD0 forms dimers in detergent micelles and most likely also in membrane although dimerization after solubilization of the membranes cannot be excluded. TMD0 dimerization observed within this study is in contrast to the results of Kamakura and coworkers detecting only monomeric TMD0^22^. However, they used a longer TMD0 construct (residue 1 to 182) and the harsher detergent polyethylenglycol(40)-nonylphenolether (NP-40). The sensitivity of protein interaction is also demonstrated by the interaction between TMD0 and coreTAPL, which is only preserved in the presence of digitonin but not with DDM.

Remarkably, TMD0 does only interact with coreTAPL but not with full length TAPL. Therefore, TMD0 does not form a dimer in the wt transport complex, which is supported by the observation that one TMD0 is sufficient to correctly localize a complex composed of wt and coreTAPL in lysosomes (Ö. Demirel, R. Abele unpublished results). Moreover, the dimerization interface of TMD0 and the binding site with coreTAPL seem to overlap. Therefore, we presume that dimerization of TMD0 protects the more hydrophilic interface from exposure to the hydrophobic surrounding of lipids or detergents. From PELDOR spectroscopy, interspin distances of roughly 3.5 nm were assigned, which fit to an antiparallel orientation of the monomers. EPR derived distances of similar range are reported for the antiparallel dimer of the small multi-drug resistant transporter EmrE from *E. coli*^23^.

Derived from chemical shifts of backbone and side chain atoms, five helices were assigned for TMD0 of TAPL with an α-helical content of 75%, which fits well to 80% determined by circular dichroism^17^. Overall, the helices match with commonly used prediction programs. However, in the experimentally determined structure, TMH1 is extended by one helical turn and TMH2 is shifted by a helical turn to the N-terminal end, thus coming closer to LH. Residues 65 to 69 adjacent to the C-terminal end of TMH2 are in contact with the hydrophobic fatty acid side chains of the detergent c6-DHPC and are mostly protected from water. The loop regions are solvent exposed as demonstrated by cross-peaks with water. Interestingly, the lysosomal lumenal end of TMH1 and the cytosolic end of TMH4 are in contact with detergent and water, which is indicating high flexibility or loose packing. All TMHs contain polar and even charged residues. To be protected by the lysosomal glycocalyx against proteolysis of abundant proteases, the lysosomal loops are short^24^. The short luminal LH forms an amphipathic helix allowing the association with the membrane. It is reported that amphipathic helices are involved in lipid sensing and shaping of organelles^25^. The TAPL orthologues Haf-4 and Haf-9 of *Caenorhabditis elegans* are essential for the formation of large gut granules^26^, and recent data suggest that human TAPL could be involved in the maturation of late phagosomes in bone marrow derived dendritic cells^27^. Therefore, it will be interesting to probe the function of the lysosomal amphipathic LH of TAPL.

The well-resolved NMR spectrum is an indication for a stable structure of the protein. However, because of the ambiguity of the restraints, all attempts to solve the three-dimensional structure involving long range NOEs, PREs, and spin distance distributions of spin probe MTSL labeled single cysteine variants of TMD0 were not successful, neither when using combined assignment and dynamics algorithm for NMR applications (CYANA), nor with a combination of chemical shift resolution adapted structural recombination (Rosetta) and ensemble refinement. This ambiguity of restraints seems to result from an ensemble of dimeric structures.

Meanwhile, single particle cryo-EM structures of ABC transporters featuring an N-terminal TMD0 have been solved. In the case of heterodimeric TAP, the TMD0s of TAP1 and TAP2 are not resolved. So far, the TMD0s could neither be analyzed alone, nor as part of the PLC in which TMD0 mediates the interaction with tapasin^7,28^. In the full-size transporters MRP1 and SUR1, the TMD0 is visible but the resolution is significantly reduced in comparison with the transmembrane domain of the core complex^15,16^. The TMD0 comprises a five TMH bundle whereas the five TMHs do not contact the core transporter. The low resolution or uncharacterized regions of the TMD0s indicate a high flexibility of these domains, and even the presence of an interaction partner does not stabilize a single conformation. Also TMD0 of TAPL could not be stabilized. Neither constructs including TMHs of coreTAPL or the interaction partner LAMP-1 nor adding the C-terminal fragment of LAMP-1 allowed to determine the structure^17^. A promising development to obtain higher resolution by reducing the dynamics will be the insertion of membrane proteins in nanodiscs. Recently, small lipid nanodiscs with a diameter of 6- to 8 nm were successfully applied for solution NMR of membrane proteins^29^. The insertion of the membrane protein can be performed by classical reconstitution procedures or co-translationally inserted by cf expression^30^. TMD0, co-translationally inserted into nanodiscs, could be used to determine the stoichiometry in lipid environment^31^. For NMR spectroscopy, a high insertion efficiency into nanodiscs has to be guaranteed.

## Materials and Methods or Experimental Procedures

### Materials

For Immunoprecipitation and detection by immunoblotting, the following antibodies were used: mouse anti-myc (monoclonal, 4A6; Millipore now Merck), mouse anti-Flag M2 (monoclonal, F1804; Sigma-Aldrich now Merck), mouse anti-His (monoclonal, HIS-1; Sigma-Aldrich now Merck), rabbit anti-TAPL^8^ and mouse anti-IgG1 (monoclonal, ab18437; Abcam); horseradish peroxidase-conjugated goat anti-rabbit (polyclonal; BD Pharmingen) and goat anti-mouse (polyclonal, A2554; Sigma-Aldrich now Merck). RRYCKSTEL peptide was synthesized by solid-phase synthesis (Charité), labeled with 5-iodoacetamidofluorescein (I9271; Sigma-Aldrich now Merck), purified by reversed-phase HPLC and verified by mass-spectrometry^32^.

### DNA constructs

Cloning of cf-TMD0-His (M_1_ – P_142_) construct of human TAPL (ABCB9, European Nucleotide Archive: AAF89993.1) in pIVex2.3-MCS (Roche) was published before^17^. Cf- TMD0-myc containing a C-terminal myc-tag (EQKLISDEEDL), Cf-TMD0-myc/His and cf-TMD0-Flag/His containing an additional myc- or Flag-tag (DYKDDDK) in front of the C-terminal His_10_-tag were generated using following primers for PCR: cf-TMD0-myc forward (5′-GATATACATATGAAATTATATAAAGCTGTTGTTGTTACTTTGGC-3′), cf-TMD0-myc reverse (5′-CGCTCGAG TCATCACAGATCCTCTTCTGAGATGAGTTTTTGTTCTGGCCGCACGGTGGAC-3′), cf-TMD0-myc/His forward (5′-CCCAAGCTGGCTAGCCATATG-3′), cf-TMD0-myc/His reverse (5′-CTCGCTCGAGTCATCAGTGATGGTGATGGTGATGGTGATGGTGATGCAGATCCTCTTCTGAGATGAGTTTTTG-3′) and cf-TMD0-Flag/His reverse (5′-CTCGCTCGAGTCATCAGTGATGGTGATGGTGATGGTGATGGTGATGCTTGTCGTCATCGTCTTTGTAGTC-3′). PCR products were cloned by NheI and XhoI into mentioned vector. Cf-TMD0-His S_28_ and S_138_ were generated by ligase chain reaction using primer pairs S_28_C (5′-CATCTATGTCTTCTGCCACCTGGACCG-3′ and 5′-GGTGGCAGAAGACATAGATGGCC GTG-3′) and S_138_C (5′-GGCTGCTGTGCACCGTGCGGC-3′ and 5′-CACGGTGCA CAGCAGCCACCAGAGC-3′) with cf-TMD0-His as template, mutated codons are underlined. For cell-based expression of wt and cf-TMD0 variants, constructs containing a C-terminal myc- or Flag-tag were generated by PCR using the primer pairs TMD0-Flag (5´-CCCAAGCTGGCTAGCATGCG-3′ and 5′-CTAGACTCGAGTCATCACTTGTCGTCATCGT CTTTGTAGTCTGGCCGCACGGTGGA C-3′), cf-TMD0-myc (5′-GATATAGCTAGCCAT ATGAAATTATATAAAGCTGTTGTTGTTACTTTGGCCTTC-3′ and 5′-GCTCGCTCGAGTCATCACAGATCC-3′) and cf-TMD0-Flag (5′-CCAAGCTGGCTAGCCATATGAAATTATATAAAGC-3′ and 5′-CTAGACTCGAGTCATCACTTGTCGTCATCGTCTTTGTAGTCT GGCCGCACGGTGGAC-3′) and were cloned by restriction sites NdeI or NheI and XhoI into the mammalian expression vector pcDNA3.1(+) (Invitrogen now Thermo Fisher). Furthermore, we used TMD0-myc, coreTAPL (MG_143_–A_766_) and TAPL (M_1_–A_766_) for cell-based expression as published before^12,13^. All used constructs (Fig. S1) were verified by DNA sequencing.

### Cell line

HEK 293 T cells were cultured at 37 °C, 5% CO_2_ and 95% humidity in Dulbecco’s Modified Eagle Medium (DMEM) (Gibco) supplemented with 10% fetal bovine serum (FCS) (Capricoron Scientific).

### Transfections

HEK 293 T cells (7 × 10^6^) were seeded in 10 cm dishes with DMEM containing 10% FCS 4 h prior to transfection. 12 µg of DNA and 48 µg of polyethylenimine (PEI) were diluted in 525 µl Opti-MEM (Gibco). For co-expression of two different genes 6 µg of DNA per construct were used. DNA and PEI solutions were mixed and incubated for 30 min at RT and dispensed carefully to the cells. Medium was exchanged 12 h after transfection with DMEM containing 10% FCS. 48 h post transfection, cells were harvested with a cell scraper, washed with ice cold PBS (Gibco) and subsequently used for immunoprecipitation experiments.

### Immunoprecipitation

For immunoprecipitation sheep anti-mouse Dynabeads (Life Technologies) were washed with 3 ml IP buffer I (20 mM Tris, 150 mM NaCl, 5 mM MgCl_2_, pH 7.4) supplemented with 0.1% bovine serum albumin (BSA) prior and after coating over night at 4 °C with mouse anti-myc, mouse anti-Flag or isotype matched non-specific mouse antibodies. Transfected HEK 293 T cells (15 × 10^6^) were harvested and solubilized in 1 ml IP buffer I containing 1% digitonin (Millipore now Merck) and 1x HP protease inhibitor mix (Serva) for 1 h at 4 °C. Cell lysate was centrifuged at 110,000 × g for 1 h at 4 °C and supernatant was incubated with antibody coated beads for 2 h at 4 °C. Beads were washed with 3 ml IP buffer I containing 0.1% digitonin and 0.5x HP protease inhibitor mix. To analyze the dynamics of TMD0 oligomerization, we used purified cf-TMD0-myc/His and cf-TMD0-Flag/His in IP buffer II (20 mM Tris, 500 mM NaCl, 5 mM MgCl_2_, pH 7.5) supplemented with 0.75% (w/v) of c6-DHPC (Avanti Polar Lipids) and 0.5x HP protease inhibitor mix. Prior to immunoprecipitation proteins were incubated up to 5 days in a molar ratio of 1:1 at 4 °C or RT. Dynabeads were washed with IP buffer II supplemented with 0.1% (w/v) BSA, coated as described before, incubated with 1 µg total protein for 2 h at 4 °C and washed with 3 ml IP buffer II supplemented with 0.75% (w/v) c6-DHPC and 0.5x HP protease inhibitor mix. All proteins were eluted in 30 µl SDS sample buffer (62.6 mM Tris, 2 mM EDTA, 1% (w/v) SDS, 0.01% (w/v) bromophenol blue, 10% (v/v) glycerol, pH 6.8) for 20 min at 90 °C. Samples were analyzed by Tricine-SDS-PAGE (10%) followed by immunoblotting^33^.

### Protein expression and purification

Cf-TMD0-His, cf-TMD0-myc, cf-TMD0-myc/His and cf-TMD0-Flag/His were produced in a continuous exchange cf *E. coli* based expression system. Solubilization was performed in LMPG (Avanti Polar Lipids), and detergent was exchanged during immobilized metal affinity chromatography (IMAC) to c6-DHPC and purified as described previously in NMR sample buffer (25 mM Na acetate, 100 mM NaCl, 0.75% (w/v) c6-DHPC, 1x HP protease inhibitor mix, pH 5.0)^17^. CoreTAPL with a C-terminal mVenus and His_10_-tag (coreTAPL_mV_) was expressed in *P. pastoris*, solubilized with DDM and purified as published before^32^. Expression, solubilization with DDM and purification of TmrAB was adapted from previously published protocols^34^ with the following modifications: cells were sonicated instead of using french press, and neither tobacco etch virus protease treatment to remove the His_10_-tag on TmrA, nor additional size exclusion chromatography have been performed.

### Reconstitution

Purified cf-TMD0-His, coreTAPL_mV_ and TmrAB were reconstituted into Triton X-100 destabilized liposomes composed of *E. coli* polar lipids and DOPC (Avanti Polar Lipids) in a 7:3 (w/w) ratio^35^. The protein-to-lipid mass ratio for reconstitution of cf-TMD0-His together with coreTAPL_mV_ or TmrAB was set to 1:20 whereas for reconstitution of solely cf-TMD0-His a 1:90 ratio was used. The molar ratio between cf-TMD0-His and dimeric coreTAPL_mV_ or TmrAB was 1:1. Detergents were removed by sequential addition (four times, minimum 1 h incubation at 4 °C) of equilibrated polystyrene beads (Bio-Beads SM-2; Bio-Rad), proteoliposomes were harvested by centrifugation for 1 h at 4 °C and 300,000 × g, and resuspended in reconstitution buffer (20 mM HEPES, 140 mM NaCl, 5% (v/v) glycerol, pH 7.5) to a final lipid concentration of 5 mg/ml and stored at −80 °C.

### Peptide transport

50 µl of proteoliposomes (1 mg/ml lipid) in transport buffer (20 mM HEPES, 107 mM NaCl, 3 mM MgCl_2_, 3 µM RRYC_Fluorescein_KSTEL, 5% (v/v) glycerol, pH 7.5) were incubated in the presence or absence of 3 mM ATP for 15 min at 37 °C. Transport was started by addition of ATP and stopped by 200 µl of stop buffer (PBS, 10 mM EDTA, pH 7.5). Proteoliposomes were collected on filter membranes (MultiScreen plates, Durapore membrane, 0.65 µm pore size; Millipore now Merck) preincubated with 0.3% (w/v) PEI, washed three times with ice-cold stop buffer and incubated for 10 min at RT with elution buffer (PBS, 1% (w/v) SDS, pH 7.5). Prior to quantification of transported peptide by a fluorescence plate reader (Polastar Galaxy, BMG) at λ_ex/em_ = 485/520 nm samples were heated for 10 min at 95 °C.

### ATP agarose pull-down

100 µl of proteoliposomes (5 mg/ml lipid) were thawed on ice, solubilized with 2% (w/v) digitonin or DDM for 1 h at 4 °C in pull-down buffer (20 mM HEPES, 140 mM NaCl, 5 mM MgCl_2_, 5% (v/v) glycerol, pH 7.5) and centrifuged at 110,000 × g for 1 h at 4 °C. Supernatant was incubated for 1 h at 4 °C in an overhead shaker with 5 mg of C_8_-coupled ATP-agarose (Sigma-Aldrich now Merck), equilibrated with 3 ml of pull-down buffer supplemented with 0.1% (w/v) digitonin or 0.05% (w/v) DDM. Beads were washed with 3 ml pull-down buffer supplemented with 0.1% (w/v) digitonin or 0.05% (w/v) DDM. Proteins were eluted in 30 µl SDS sample buffer supplemented with 250 mM DTT for 20 min at 65 °C followed by Tricine-SDS-PAGE (10%) and immunoblotting.

### SEC-MALS

Size exclusion chromatography coupled with multi-angle light scattering (SEC-MALS) was performed at 4 °C using a TSK-GEL G3000SWXL column (15 ml, Tosoh Bioscience), a light-scattering detector (TREOS) and refractometer (Optilab rEX) from Wyatt Technology and a UV detector, HPLC pump and degasser from Jasco. System was equilibrated with 3 column volumes of SEC buffer (25 mM Na acetate, 300 mM NaCl, 0.7% (w/v) c6-DHPC, 1x HP protease inhibitor mix, pH 5.0) filtered through 0.1 µm pore size VVLP filters (Millipore now Merck) following a recirculation through the system for 12 h at 0.5 ml/min to improve the baseline by removing air bubbles and particles by degasser and pre-injection filter (0.1 µm). Per measurement 400 µg of protein in 200 µl SEC buffer were injected and analyzed at a flow rate of 0.5 ml/min, UV detector sensitivity was set to 3 AU = 1 V. Light scattering detector was calibrated by using monomeric BSA (Sigma-Aldrich now Merck). The obtained signals were processed with the ASTRA software package version 5.3.4.11 (Wyatt Technology). To calculate the molecular mass all three detectors were used. A refractive index increment dn/dc of 0.185 and 0.135 ml/g for proteins and c6-DHPC, respectively, and an extinction coefficient ε_280_ of 45950 [M^−1^ * cm^−1^] for cf-TMD0-His was applied.

### Site directed spin labeling

Solubilization, purification and detergent exchange to c6-DHPC of single cysteine containing cf-TMD0-His constructs S_28_C and S_138_C were performed in the presence of 1 mM DTT. After detergent exchange during IMAC, column was washed with 10 column volumes of TBS buffer (20 mM Tris, 500 mM NaCl, 20 mM imidazole, 0.75% (w/v) c6-DHPC, pH 7.5) to remove DTT. Subsequently, Ni-NTA-agarose was incubated with 10 column volumes TBS buffer supplemented with 1.4 mM MTSL (Toronto Research Chemicals) for 1 h at RT in an overhead shaker followed by an additional washing step with 10 column volumes of TBS buffer. Further purification was performed as mentioned before. Labeling efficiencies were determined by cw-EPR in NMR sample buffer by spin counting using MTSL as reference. Labeling efficiencies were between 95 and 100%.

### PELDOR spectroscopy

For PELDOR measurements, 15–20 μl of cf-TMD0-His S_28_C or S_138_C sample containing 15–20% (v/v) deuterated glycerol was transferred into 1.6 mm outer diameter quartz EPR tubes (Suprasil, Wilmad LabGlass) and quick-frozen in liquid nitrogen. Pulsed EPR data were recorded on an ELEXSYS E580 EPR spectrometer (Bruker), which is equipped with a PELDOR unit (E580-400U, Bruker), a continuous-flow helium cryostat (CF935, Oxford Instruments), and a temperature control system (ITC 502, Oxford Instruments). Measurements were performed at Q-band frequencies (33.4 GHz) using an ELEXSYS SuperQ-FT accessory unit and a Bruker AmpQ 10 W amplifier in a Bruker EN5107D2 cavity at 50 K. The dead-time free four-pulse sequence with phase-cycled π/2-pulse was used for PELDOR measurements^36^. A 20 ns pump pulse was used, which was placed at the maximum of the echo-detected field swept spectrum. The observer pulse lengths were set to 32 ns (π/2 and π), which were set 70 MHz lower. The deuterium modulations were averaged by increasing the first interpulse delay by 16 ns for 8 steps. The normalized primary PELDOR data V(t)/V(0) were processed to remove background contribution, and the resulting form factors F(t)/F(0) were fitted with a model-free Tikhonov regularization to distance distributions with DeerAnalysis2016 software package^37^.

### NMR spectroscopy

All NMR experiments were recorded at 323 K on Bruker spectrometers equipped with cryogenic ^1^H[^13^C/^15^N] triple-resonance probes with static fields ranging from 16.4 to 22.3 T (700 to 950 MHz proton frequency). Backbone ^1^H, ^15^N, ^13^C′, ^13^CA, ^1^HA and side-chain ^13^CB resonances were assigned using three dimensional ^1^H-detected [^15^N, ^1^H]-BEST-TROSY type 3D HNCO, HN(CA)CO, HNCA, HN(CO)CA, HNCACB and ^13^C-detected HCACO experiments on ^13^C/^15^N fully labeled cf-TMD0-His in conjunction with a combinatorial triple-selective labeling protocol^18^. Spectra processing and analysis were performed with TopSpin 3.2 (Bruker) and NMRFAM-SPARKY 3.13^38^. Backbone and side chain chemical shifts were used to predict secondary structure by TALOS-N^39^. For paramagnetic relaxation enhancement experiments a 100 mM stock of 16-DSA in water-free dimethylsulfoxid (DMSO) was added to 200 µM of ^15^N labeled cf-TMD0-His in NMR sample buffer to a final concentration of 1 mM. [^15^N, ^1^H]-BEST-TROSY experiments were performed, and the ratio between the peak intensity in the presence and absence of 16-DSA was determined for each residue. For detection of sequential NOEs, intermolecular NOEs with the detergent c6-DHPC and amide exchange with water a 3D NOESY-BEST-[^15^N, ^1^H]-TROSY (mixing time 300 ms) of 600 µM ^2^H and ^15^N uniformly labeled cf-TMD0-His (except for non deuterated ^15^N Asn) was recorded.

## Electronic supplementary material

Supplementary information
