# Supplementary material for: Structural and functional insights into the interaction and targeting hub TMD0 of the polypeptide transporter TAPL
Source: Sci Rep. 2018 Oct 23;8:15662. doi: 10.1038/s41598-018-33841-w (PMC6199259; doi:10.1038/s41598-018-33841-w)
Supplement: Supplementary file 1 — Supplementary information [file 41598_2018_33841_MOESM1_ESM.pdf]

Supplementary information

Structural and functional insights into the interaction and targeting hub TMD0 of the polypeptide transporter TAPL

Christoph Bock[1], Frank Löhr[2], Franz Tumulka[1], Katrin Reichel[3], Julia Würz[2], Gerhard Hummer[4], Lars Schäfer[5], Robert Tampé[1], Benesh Joseph[3], Frank Bernhard[2], Volker Dötsch[2], Rupert Abele[1*]

[1]Institute of Biochemistry, Biocenter, Goethe University Frankfurt, Max-von-Laue Str. 9, 60438 Frankfurt am Main, Germany

[2]Institute of Biophysical Chemistry & Center for Biomolecular Magnetic Resonance, Goethe University Frankfurt, Max-von-Laue Str. 9, 60438 Frankfurt am Main, Germany

[3]Institute of Physical and Theoretical Chemistry, Goethe University Frankfurt, Max-von-Laue Str. 9, 60438 Frankfurt am Main, Germany

[4]Department of Theoretical Biophysics, Max Planck Institute of Biophysics, Max-von-Laue Str. 3, 60438 Frankfurt am Main, Germany

[5]Lehrstuhl für Theoretische Chemie, Ruhr-University Bochum, 4780 Bochum, Germany

[*]Correspondence should be addressed to R.A.: abele@em.uni-frankfurt.de

Supplementary material

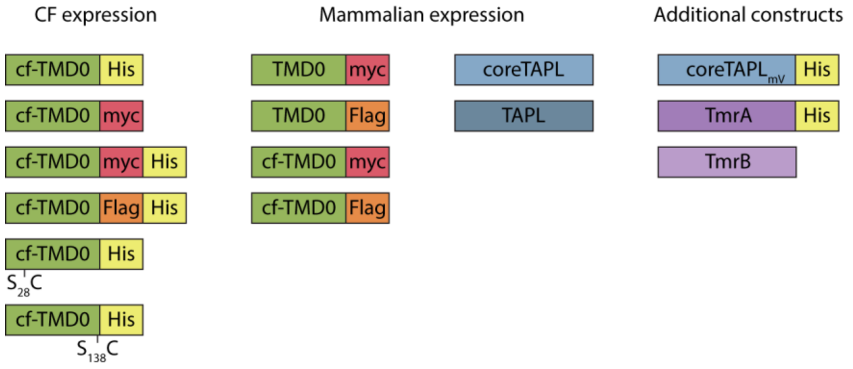

Figure S1: Expression constructs used in this study

Besides wt TMD0 (M_1 - P_{142}) a cf expression version of TMD0 (cf-TMD0) was used, in which arginine at position two was replaced by lysine, and tryptophan at position four was substituted by tyrosine. Moreover, all four cysteines were replaced by alanine. For *E. coli* based cf expression system, four different tagged variants were generated: cf-TMD0-His contains a C-terminal His_{10}-tag, cf-TMD0-myc a C-terminal myc-tag (EQKLISEEDL), cf-TMD0-myc/His and cf-TMD0-Flag/His contain an additional myc- or Flag-tag (DYKDDDDK) in front of the His_{10}-tag. Furthermore two single cysteine versions of cf-TMD0-His were used, in which serine at position 28 or 138 was replaced by cysteine. For mammalian expression in HEK 293T cells, C-terminal myc- or Flag-tagged TMD0 and cf-TMD0 were used as well as coreTAPL (G_{143} - A_{766}) and TAPL (M_1 - A_{766}). $CoreTAPL_{mV}$ with a C-terminal mVenus followed by His_{10}-tag was used for expression in *P. pastoris*, heterodimeric TmrA with a C-terminal His_{10}-tag and TmrB were used for expression in *E. coli*.

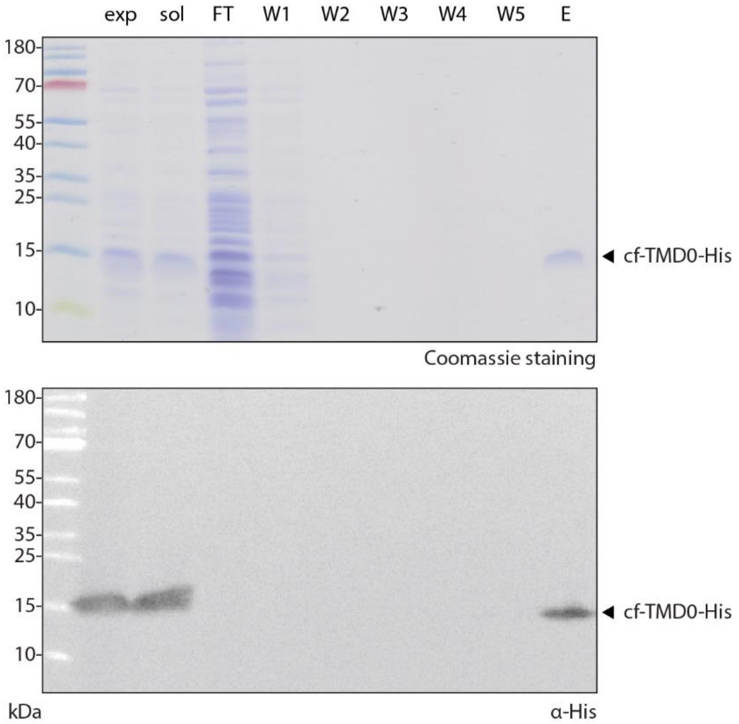

Figure S2: Solubilization, purification and refolding of cf-TMD0-His

Cf-TMD0-His was expressed in an *E. coli* based cf expression system in the absence of detergent (exp). Precipitate was solubilized with 1% LMPG (sol) for 2 h at room temperature, and flow through (FT) was collected after binding to Ni-NTA-agarose for 2 h at room temperature. For refolding, detergent was exchanged during IMAC purification in 5 consecutive washing steps from LMPG to 0.75% c6-DHPC (W1 - W5) prior to elution and buffer exchange to NMR sample buffer (E). Purification was analyzed by Tricine-SDS-PAGE (10%) followed by Coomassie staining (top) or immunoblotting with His-tag specific antibody (bottom). Identical aliquots are loaded except flow through and washing steps, which were loaded in a 10-fold excess.

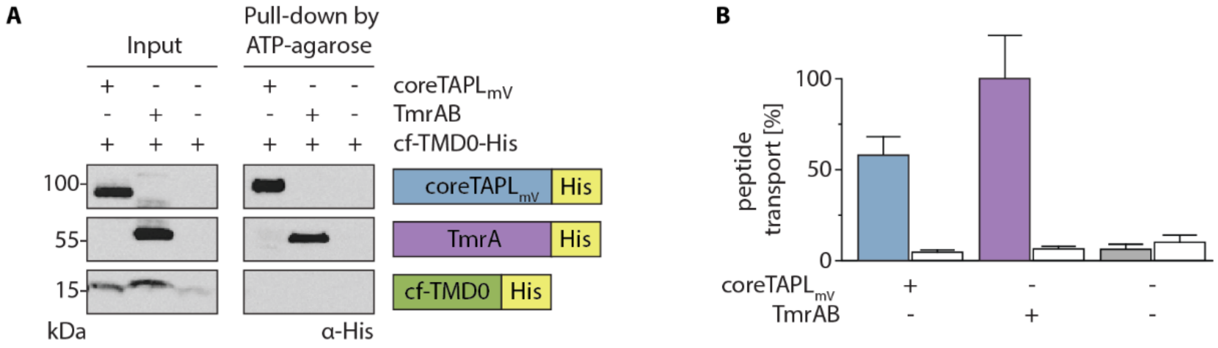

Figure S3: DDM disrupts interaction between TMD0 and coreTAPL

(**A**) After reconstitution of coreTAPL$_{mV}$, TmrAB and cf-TMD0-His (input) into liposomes, proteoliposomes were solubilized with 2% DDM. CoreTAPL$_{mV}$ and TmrAB were pulled-down by ATP-agarose and analyzed by immunoblotting using a His-tag specific antibody. Original, uncropped immunoblots are shown in supplementary information page 13. **(B)** Functional reconstitution was demonstrated by incubation of proteoliposomes containing only cf-TMD0-His or cf-TMD0-His and coreTAPL$_{mV}$ or TmrAB in the presence (filled bar) or absence (open bar) of ATP (3 mM) with peptide (3 µM) for 15 min at 37 °C. Transport was performed in triplicates and normalized, error bars indicated SD.

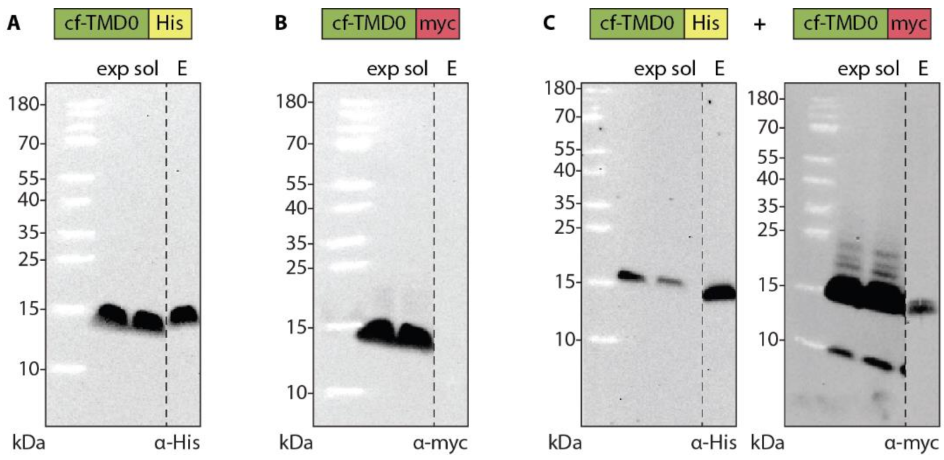

Figure S4: His-tag independent oligomerization

To demonstrate His-tag independent oligomerization of cf expressed TMD0, cf-TMD0-His and cf-TMD0-myc were separately (**A** and **B**) or co-expressed (**C**) by the *E. coli* based cf expression system (exp). Cf-TMD0 variants were solubilized in LMPG (sol), purified by Ni-NTA-agarose, reconstituted in c6-DHPC, eluted from the column by imidazole (E) and analyzed by immunoblotting. Original, uncropped immunoblots are shown in supplementary information page 13 and 14.

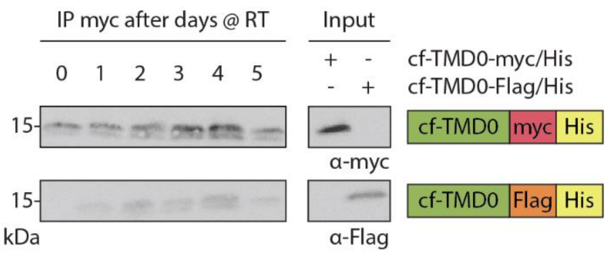

Figure S5: Dynamics of TMD0 oligomers at room temperature

To probe oligomerization dynamics, cf expressed TMD0 variants were separately purified and refolded (input). Cf-TMD0-myc/His and cf-TMD0-Flag/His were mixed in a 1:1 molar ratio and incubated at RT up to five days. Exchange of subunits was analyzed by immunoprecipitation with myc-tag specific antibody. Input represents 1/15 aliquot of the volume used for pull-down. Original, uncropped immunoblots are shown in supplementary information page 14.

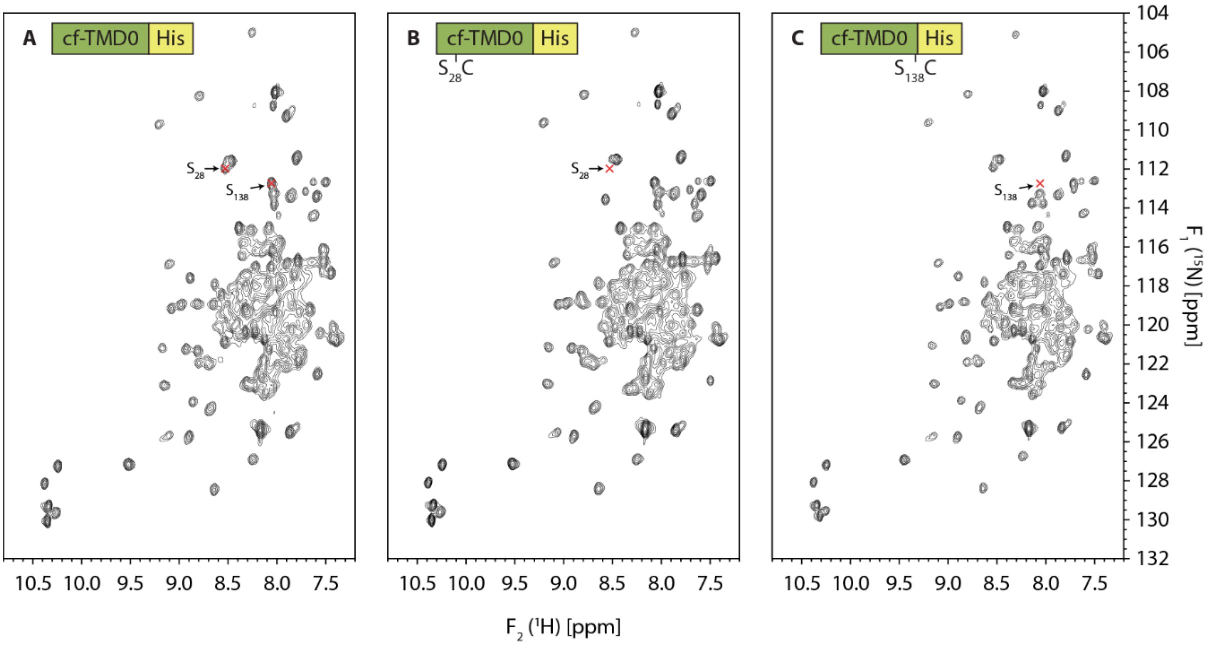

Figure S6: Structural integrity of cf-TMD0-His $S_{28}C$ and $S_{138}C$

2D-[^{15}N, ^{1}H]-TROSY NMR spectra were recorded of ^{15}N uniformly labeled (**A**) cf-TMD0-His (160 µM, 700 MHz), **(B)** cf-TMD0-His $S_{28}C$ (140 µM, 700 MHz) and (**C**) cf-TMD0-His $S_{138}C$ (180 µM, 700 MHz) at 323 K in 25 mM Na-acetate, 100 mM NaCl and 0.75% c6-DHPC, 1x HP protease inhibitor mix, pH 5.0. NMR sample buffer for single cysteine mutants was supplemented with 1 mM DTT. Peaks for mutated serine residues are indicated by a red cross.

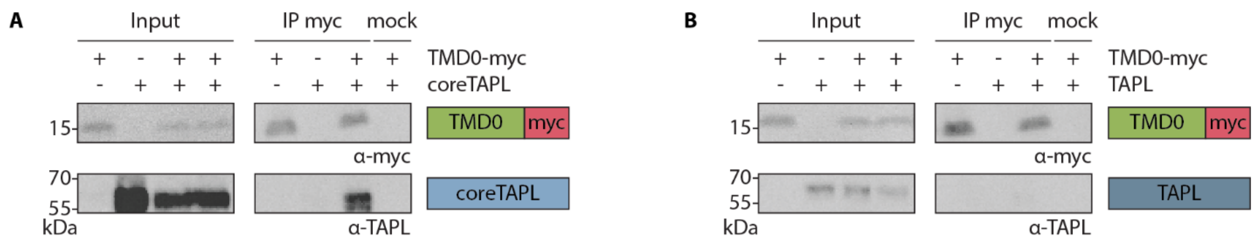

Figure S7: TMD0 interaction with coreTAPL and TAPL

TMD0-myc was transiently co-expressed with coreTAPL (**A**) or TAPL (**B**) in HEK 293T cells (input). Proteins were solubilized by 1% digitonin, immunoprecipitated by myc-tag specific antibody (IP myc) or isotype control (mock) and analyzed by immunoblotting. Input represents 1/25 aliquot of the volume used for immunoprecipitation. Original, uncropped immunoblots are shown in supplementary information page 15.

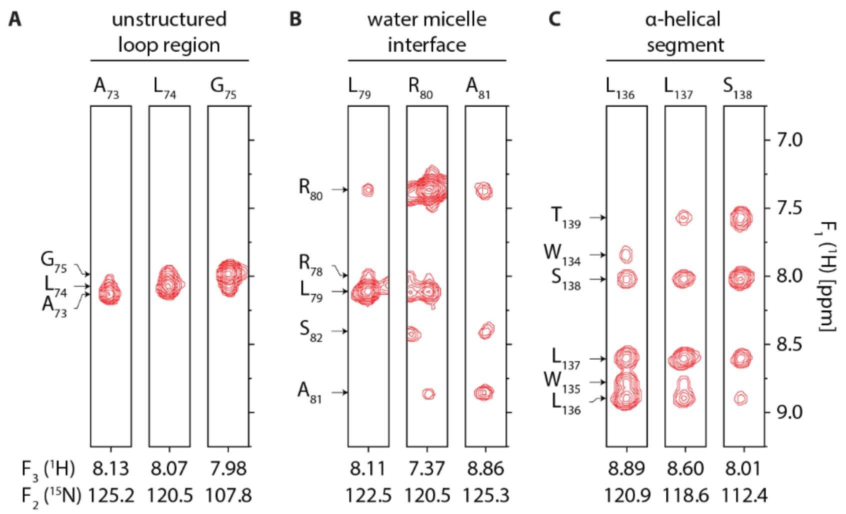

Figure S8: Sequential backbone NOEs in TMD0

Representative spectral sections of unstructured loop region (**A**), water micelle interface (**B**) and α-helical segment (**C**) from a 3D NOESY-BEST-[^{15}N, ^{1}H]-TROSY experiment (950 MHz, mixing time 300 ms) at 323 K of ^{2}H partially and ^{15}N uniformly labeled cf-TMD0-His (600 μM). Sections are taken at F_2 (^{15}N) chemical shifts and centered (0.1 ppm) at the ^{1}H chemical shifts along F_3. Assignments of amide-amide NOE contacts detected along the ^{1}H (F_1) axis are indicated.

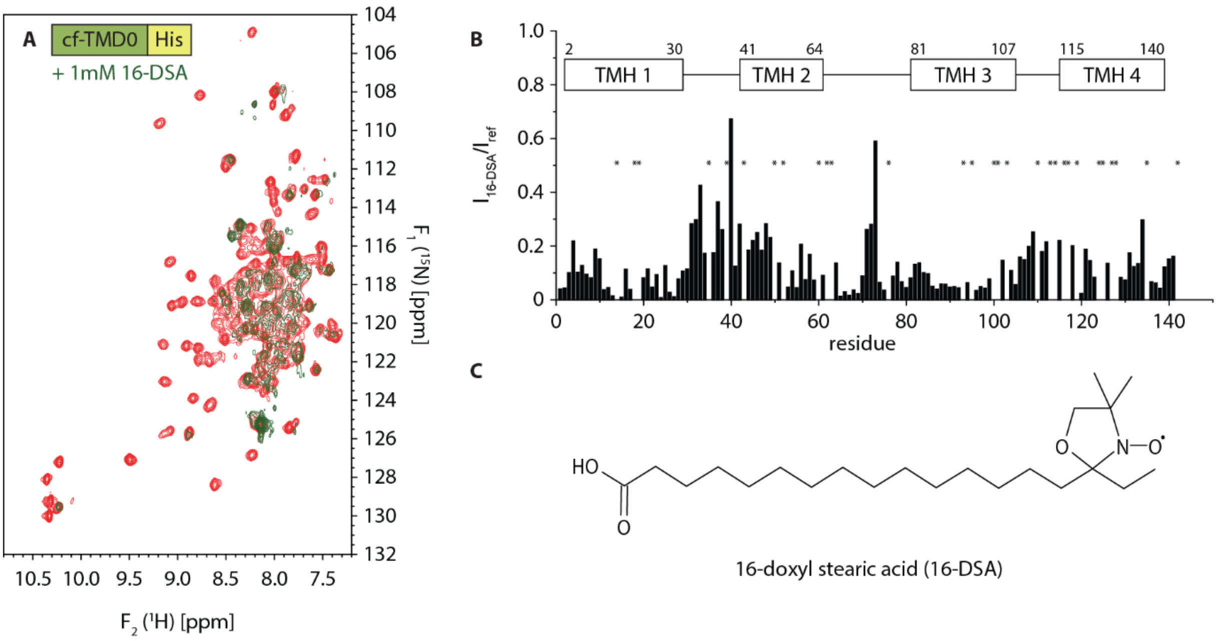

Figure S9: Paramagnetic relaxation enhancement of cf-TMD0-His by 16-DSA

(**A**) 2D-[^{15}N, ^{1}H]-TROSY NMR spectra (800 MHz) of ^{15}N uniformly labeled cf-TMD0-His (200 µM) in the absence (red) or presence (green) of 1 mM 16-DSA were recorded at 323 K in 25 mM Na-acetate, 100 mM NaCl, 0.75% c6-DHPC and 1x HP protease inhibitor mix, pH 5.0. (**B**) Paramagnetic relaxation enhancement was plotted for each residue as ratio of the peak intensities in the presence and absence of 1 mM 16-DSA. Stars indicate ratios of zero resulting from spectral overlap or incomplete backbone assignment. **(C)** Structure of 16-DSA.

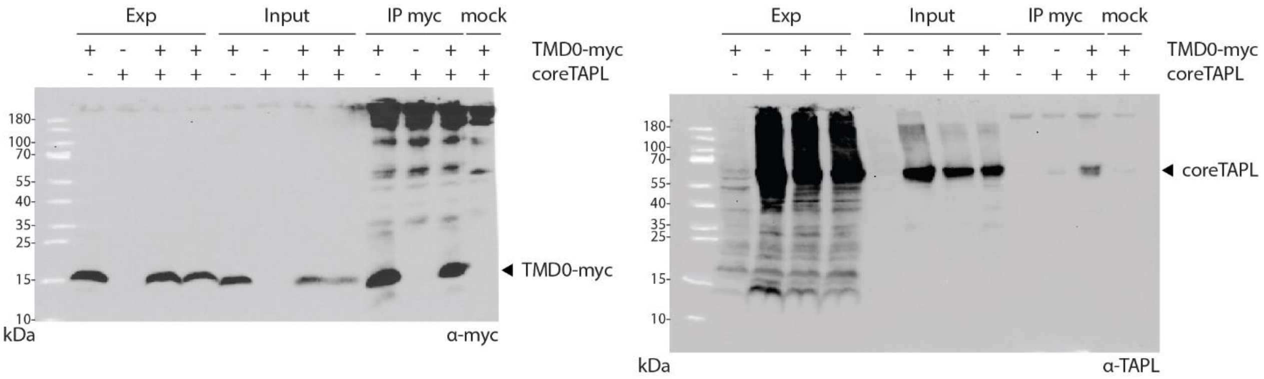

Figure 2A: Uncropped immunoblots

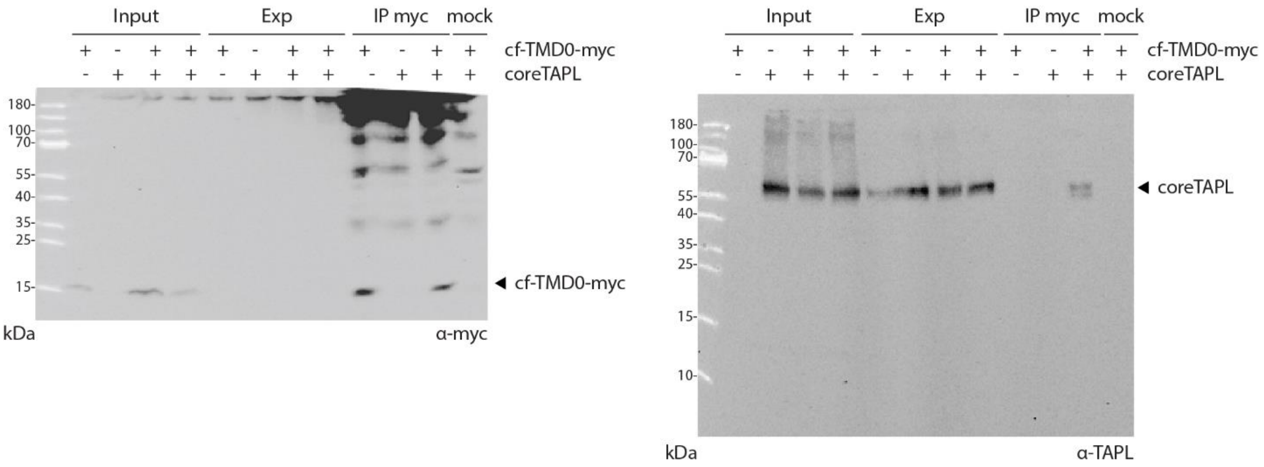

Figure 2B: Uncropped immunoblots

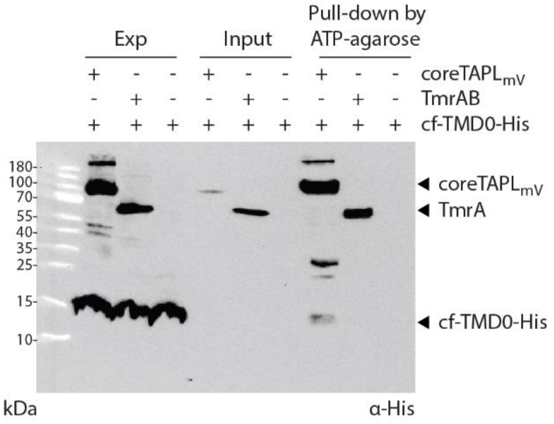

Figure 2C: Uncropped immunoblots

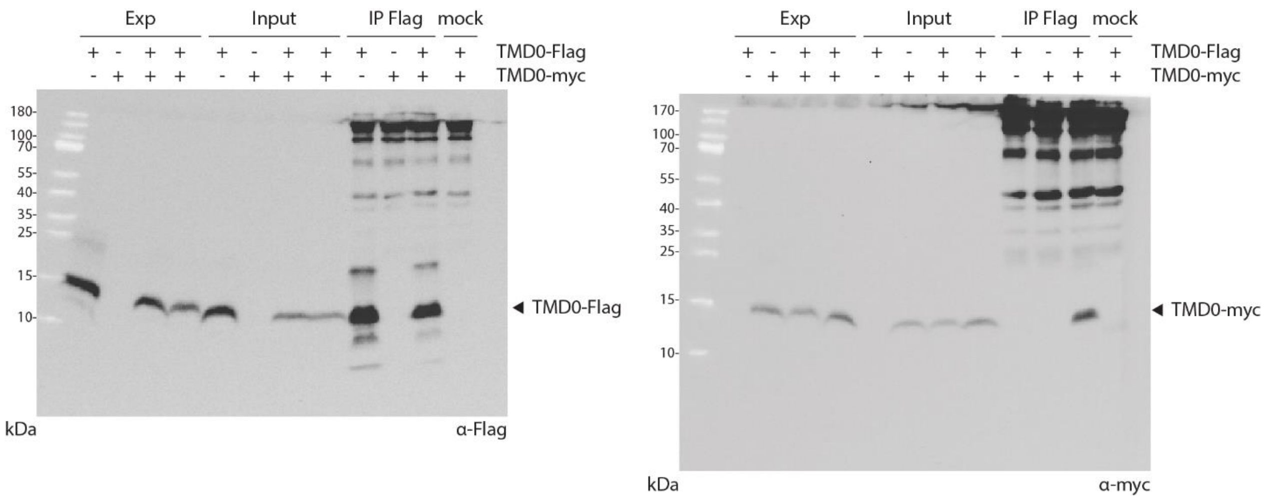

Figure 3A: Uncropped immunoblots

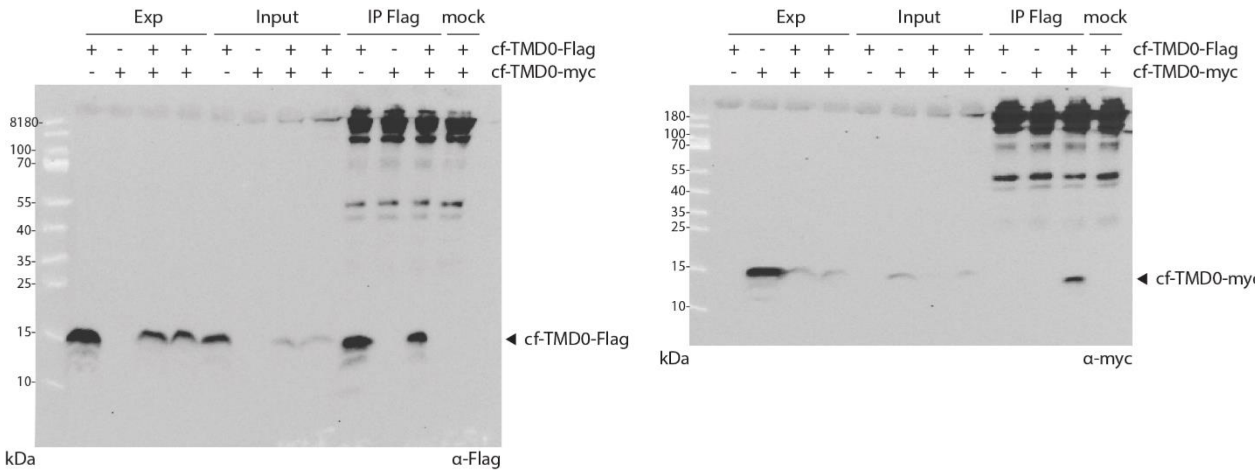

Figure 3B: Uncropped immunoblots

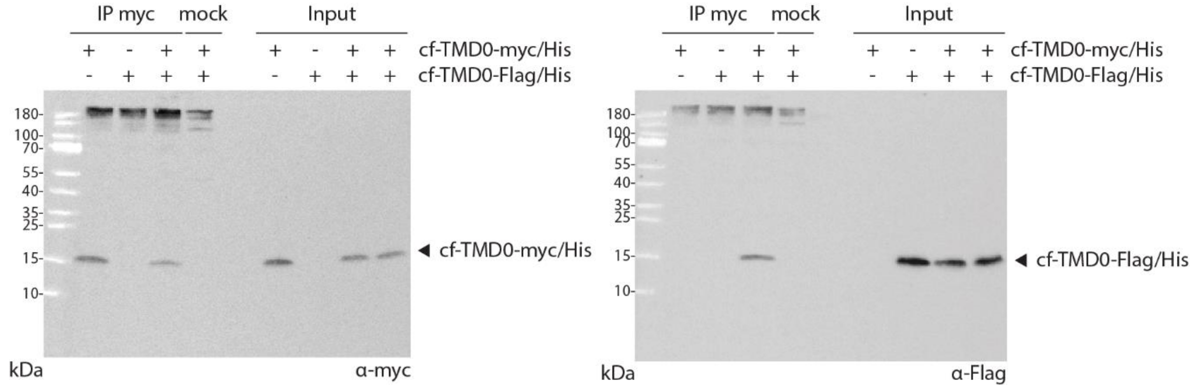

Figure 3C: Uncropped immunoblots

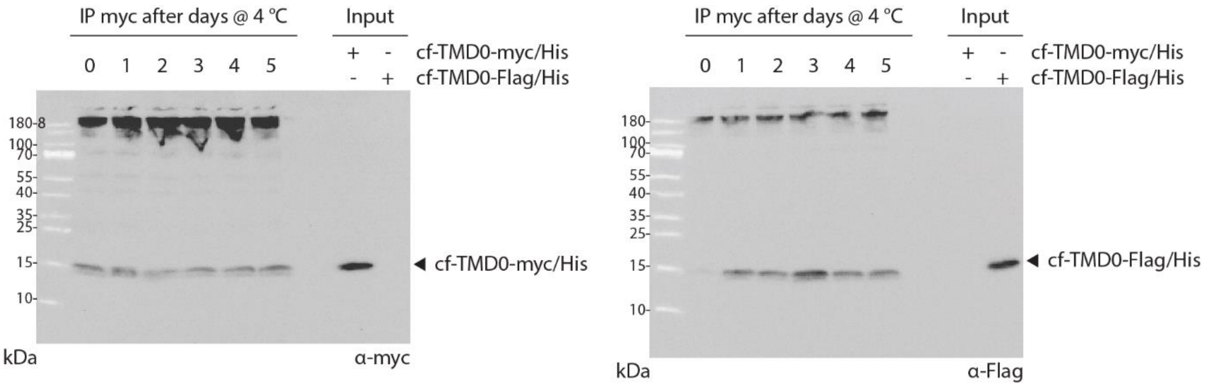

Figure 3D: Uncropped immunoblots

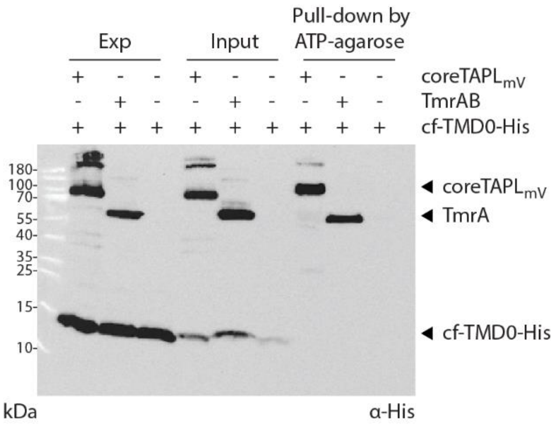

Figure S3A: Uncropped immunoblots

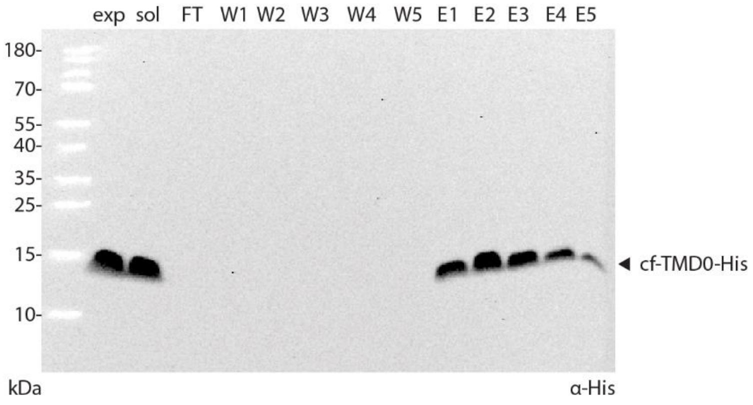

Figure S4A: Uncropped immunoblots

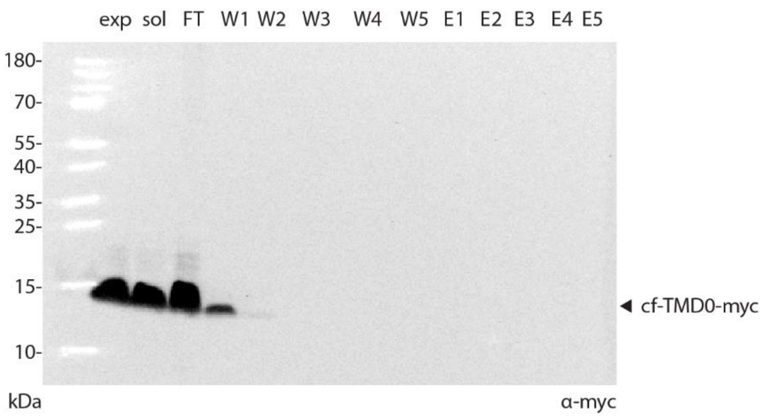

Figure S4B: Uncropped immunoblots

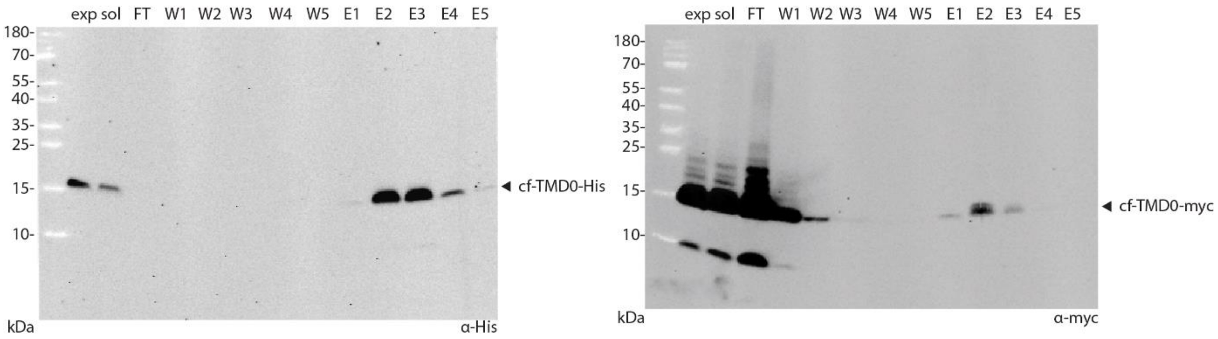

Figure S4C: Uncropped immunoblots

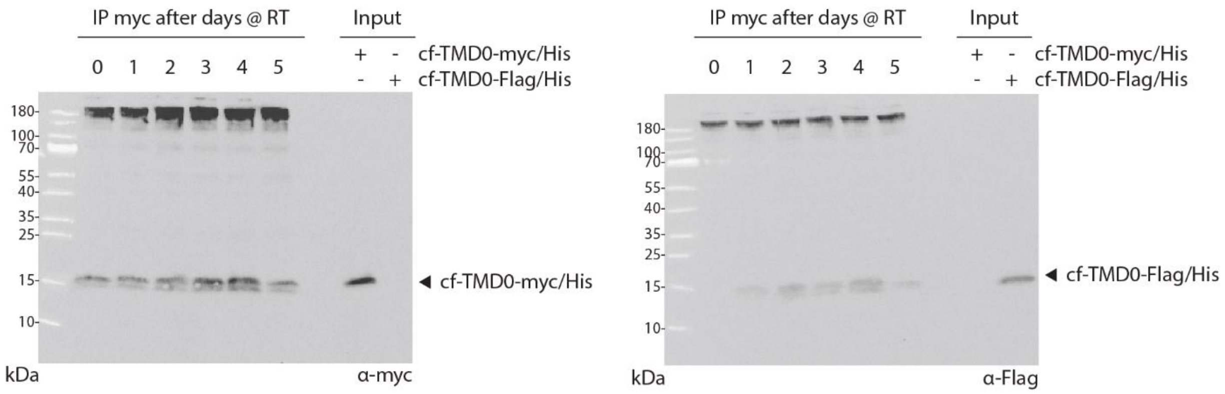

Figure S5: Uncropped immunoblots

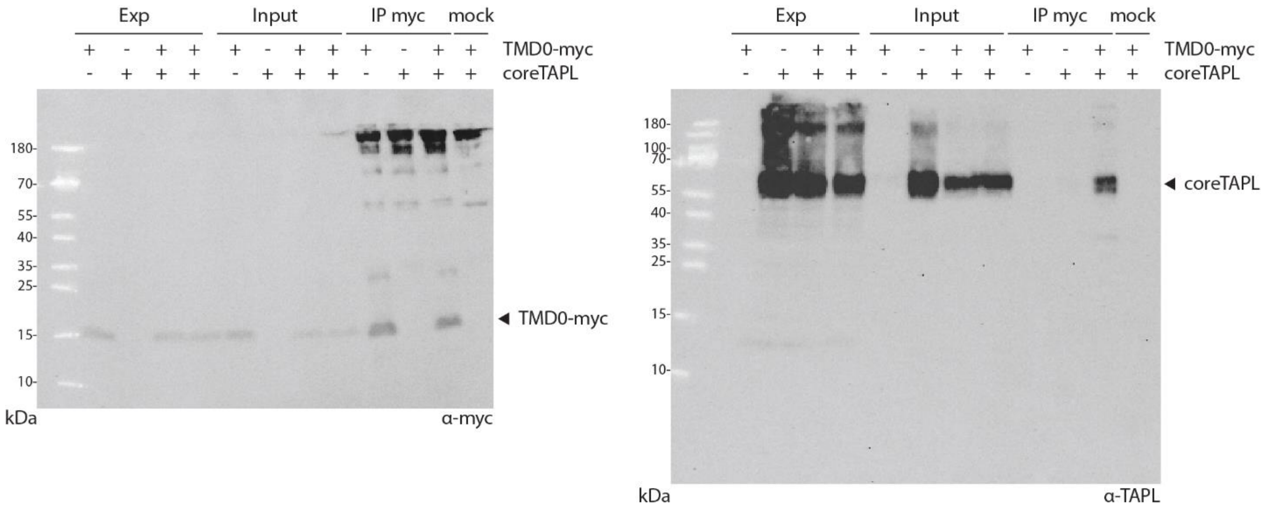

Figure S7A: Uncropped immunoblots

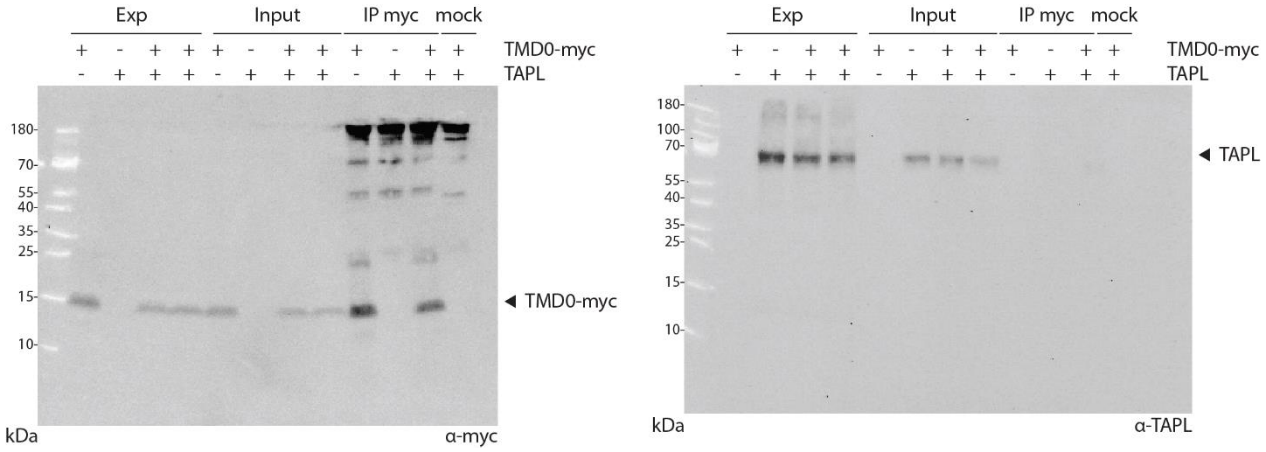

Figure S7B: Uncropped immunoblots
